# The XGBoost Model Versus the Logistic Regression Model Created Based on Serum Markers in Predicting the Risk of Post‐Stroke Cognitive Impairment Following Acute Ischemic Stroke

**DOI:** 10.1002/brb3.71373

**Published:** 2026-04-15

**Authors:** Xiaofeng Yang, Xianwen Li, Yanfeng Wu, Qian Zhang, Juan Chen, Fengjiao Zhao

**Affiliations:** ^1^ Department of Neurology Second Affiliated Hospital of Nanjing Medical University Nanjing Jiangsu China; ^2^ School of Nursing Nanjing Medical University Nanjing Jiangsu China; ^3^ Department of Neurology Nanjing Drum Tower Hospital Affiliated to Nanjing University Medical School Nanjing Jiangsu China

**Keywords:** acute ischemic stroke, logistic regression, post‐stroke cognitive impairment, serum markers, XGBoost

## Abstract

**Background**: Acute ischemic stroke is a major cause of cognitive dysfunction. Early identification of post‐stroke cognitive impairment (PSCI) is crucial for improving patient prognosis. While there has been extensive research on prognostic models for acute ischemic stroke, the selection of predictive factors remains heavily reliant on neuroimaging parameters. This study aims to create and compare the eXtreme gradient boosting (XGBoost) and logistic regression (LR) models based on serum biomarkers for predicting the risk of PSCI following acute ischemic stroke.

**Methods**: The study enrolled 261 adult patients with acute ischemic stroke within 7 days of onset. Their baseline characteristics, serum markers, and scores anthe National Institutes of Health Stroke Scale (NIHSS) and the Montreal Cognitive Assessment (MoCA) were collected. Cognitive function assessment was completed 3 months (±2 weeks) after stroke, with PSCI diagnosis based on a MoCA score < 26. Patients were randomly assigned to the training dataset (*n* = 183) and testing dataset (*n* = 78) in a ratio of 7:3. Significant features for predicting the risk of PSCI were selected via LassoCV in R. The accuracy, F1 score, Cohen's kappa, sensitivity, specificity, positive predictive value (PPV), and negative predictive value (NPV) were measured to assess the accuracy of the XGBoost and LR prediction models. Finally, the performance of the optimal prediction model was evaluated by SHapley additive exPlanations (SHAP) beeswarm and force plots.

**Results**: The incidence of PSCI and other baseline characteristics were comparable between the training and testing datasets (all *P* > 0.05). Vascular endothelial cadherin (VE‐Cad), NIHSS score, age, drink history, C‐reactive protein (CRP), and education years were features associated with the risk of PSCI. The XGBoost model was superior in accuracy, F1 score and sensitivity in predicting the risk of PSCI than the LR model. Beeswarm and force plots displayed the excellent ability of the XGBoost model in predicting the risk of PSCI in patients with acute ischemic stroke.

**Conclusion**: Based on serum biomarkers, the XGBoost model can accurately predict the risk of PSCI in patients with acute ischemic stroke, with superior performance than the LR model, and may serve as a reliable tool for early identification to improve the diagnosis.From 261 acute ischemic stroke patients (training *n* = 183, testing *n* = 78), we collected demographic data, cognitive assessments, and serum indicators. LassoCV identified sensitive predictors including VE‐Cad, NIHSS score, CRP, age, drinking history, and education years. The XGBoost model demonstrated superior performance over LR in predicting PSCI risk. SHAP analysis revealed how these variables influenced model predictions. Based on serum biomarkers, the XGBoost model accurately predicts PSCI risk and may serve as a reliable tool for early identification to improve diagnosis.

## Graphical abstract

From 261 acute ischemic stroke patients (training n = 183, testing n = 78), we collected demographic data, cognitive assessments, and serum indicators. LassoCV identified sensitive predictors including VE‐Cad, NIHSS score, CRP, age, drinking history, education years. The XGBoost model demonstrated superior performance over logistic regression in predicting PSCI risk. SHAP analysis revealed how these variables influenced model predictions. Based on serum biomarkers, the XGBoost model accurately predicts PSCI risk and may serve as a reliable tool for early identification to improve diagnosis.

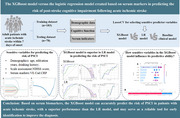

## 1 Introduction

Acute ischemic stroke is a severe disease characterized by high rates of incidence, mortality, disability, and recurrence. Survivors usually suffer from a wide range of long‐term sequelae, including motor dysfunction, language deficits, and cognitive impairment. Post‐stroke cognitive impairment (PSCI), as a common complication of stroke within 6 months, affects 24%–53.4% of these survivors (Liu et al. 2019; Lo et al. 2019). Directly curbing the activities of daily life and the rehabilitation process, it poses a huge economic burden on affected people and their families (Lo et al. 2019; Xue et al. 2021). PSCI is an intermediate stage between post‐stroke non‐dementia and dementia, and effective approaches to reverse it are currently scant. PSCI might recover due to rehabilitation and neuroplasticity during the first few months after stroke, while less improvement would be found after 6 months (Ma et al. 2024). As a result, early recognition and treatment of PSCI are of great significance to improve the prognosis.

So far, the exact pathophysiological mechanism of PSCI has not yet been fully elucidated. PSCI is generally considered as outcome of direct damage to key brain areas involved in cognition (e.g., frontal and temporal lobes). However, a complex interplay involving more than just stroke location and volume drives the heterogeneity and progression of PSCI (Sun et al. 2014). Existing evidence pinpoints that PSCI is a dynamic cascade triggered by acute vascular events, which evolves via multiple mechanisms like neuroinflammation, blood‐brain barrier (BBB) disruption, neurodegeneration, and remodeling of whole‐brain functional connectivity (Khan et al. 2025; Sagues et al. 2025). Enveloped in mystery, unidentified interactions, temporal relations, and dominant factors involved in possible pathophysiological mechanisms of PSCI directly hinder the design and development of targeted interventions (Sagues et al. 2025). Demographic and neuroimaging data are valuable for the prediction of PSCI (Kandiah et al. 2016; Chander et al. 2017; Munsch et al. 2016; Coutureau et al. 2021; Dong et al. 2021). However, neuroimaging is a series of expensive examinations of the brain with strict indications, and there still lacks a consensus on the neuroimaging features suitable for PSCI‐prediction modeling. While being recognized for their high accuracy, conventional plasma biomarkers for PSCI, like neurofilament light chain (NfL) and phosphorylated Tau (p‐Tau) have not been extensively applied to clinical settings for predicting PSCI due to the advanced detection techniques and high costs (Li et al. 2025). Markers of inflammation or metabolism, although highly accessible, are less specific to accurately capture the complex and dynamic changes in the acute phase of a stroke (Jiang et al. 2025; Li et al. 2025). In the present study, we aim to identify accessible serum markers of PSCI. Following a definite biological framework, PSCI biomarkers with a close relation to systemic vascular risks and metabolic disorders, stress and inflammatory responses in the acute phase of a stroke, and vascular endothelial dysfunction and multi‐organ interaction were screened. First of all, indicators of systemic vascular risks and metabolic disorders, such as hemoglobin A1c (HbA1c), blood lipids, uric acid (UA), and homocysteine (HCY), were monitored to assess blood sugar control, atherosclerosis burden, and oxidative stress in adult patients with acute ischemic stroke (Jiang et al. 2025; Li et al. 2025). Second, C‐reactive protein (CRP) was detected to sensitively reflect systemic inflammatory response (Jiang et al. 2025). Third, we innovatively analyzed vascular endothelial cadherin (VE‐Cad), fibroblast growth factor 21 (FGF21), and the blood urea nitrogen (BUN)/serum creatinine (SCr) ratio to directly capture dynamic changes in endothelial barrier integrity disruption (Nakano‐Doi et al. 2018), endogenous neuroprotective response (Wang et al. 2025), and systemic hemodynamic status (Wang et al. 2024), respectively. We then created a series of models based on an integration of serum markers, common laboratory testing indicators, and multi‐dimensional clinical factors for predicting the risk of PSCI following acute ischemic stroke, and compared their performance, aiming to offer a useful tool to simultaneously screen high‐risk individuals and guide stratified prevention strategies against PSCI.

## 2 Methods

### 2.1 Participants

On the premise that baseline assessments and capture of serum signals associated with initial damage and early pathophysiological responses were similarly conducted in all participants during the acute phase of a stroke, adult patients with acute ischemic stroke within 7 days of onset admitted to the Neurology Department, the Second Affiliated Hospital of Nanjing Medical University, from July 2023 to May 2024 were enrolled in this study. A diagnosis of ischemic stroke was made based on the Chinese Guidelines for the Diagnosis and Treatment of Acute Ischemic Stroke 2018. Excluded were those with obvious cognitive impairment comprehensively determined by structured interviews of primary caregivers and medical records before the onset of acute ischemic stroke; hemorrhagic stroke; active infections within four weeks of stroke onset requiring systemic antibiotic, antiviral, or antifungal therapies regardless of hospitalization; existing infectious fever (a maintained temperature of 38.0°C or greater) with elevated white blood cell count (>10.0 × 10^9^/L) or CRP (>10 mg/L) unrelated to stroke; benign or malignant tumors; organ dysfunction (cardiac dysfunction determined by the New York Heart Association [NYHA] Functional Classification of Class III–IV, or left ventricular ejection fraction < 40%; liver dysfunction determined by Child‐Pugh Class B–C, or severe liver damage indicated by laboratory testing of total bilirubin levels exceeding 3 times the upper limit of normal and prothrombin time prolonged by more than 6 s; renal dysfunction determined by the estimated glomerular filtration rate of lower than 30 mL/min/1.73 m^2^, or on maintenance dialysis; respiratory dysfunction determined by the demand for long‐term home oxygen therapy or mechanical ventilation; terminal diseases (e.g., malignancies); or life expectancy < 6 months); Alzheimer's disease or other non‐vascular dementia; and mental illnesses, drug/alcohol abuse, or delirium that failed the participant to cooperate. This study was approved by the Ethics Committee of the Second Affiliated Hospital of Nanjing Medical University, and written informed consent was provided by all participants or their family members. Finally, a total of 261 eligible patients with acute ischemic stroke were enrolled, involving 191 male and 70 female patients, with an average age of 66.60 ± 10.94 (41–88) years.

### 2.2 Data Collection

Demographic data, including age, gender, education level, body mass index (BMI), smoking, drinking, and history of underlying diseases (e.g., hypertension, diabetes mellitus), were collected. Scale assessments on admission were performed using the National Institutes of Health Stroke Scale (NIHSS), the Montreal Cognitive Assessment (MoCA), and the Mini‐Mental State Examination (MMSE).

A blood test was performed in fasting patients within 24 h of onset for measuring VE‐Cad, FGF21, CRP, total cholesterol (TCH), triglycerides (TG), high‐density lipoprotein cholesterol (HDL‐C), low‐density lipoprotein cholesterol (LDL‐C), HCY, UA, HbA1c, and the BUN/SCr ratio. Specifically, VE‐Cad and FGF21 were detected in serum samples harvested by a 10 min centrifugation of peripheral blood at 3000 r/min by an enzyme‐linked immunosorbent assay (ELISA) using commercial kits (Elabscience, Wuhan, China).

### 2.3 Assessment of Cognitive Function

Cognitive function was predominantly assessed by the MoCA on admission and three months post‐stroke (±2 weeks of follow‐up time window), with the assistance of the MMSE to check for overall cognitive impairment. The MoCA is a 30‐point scale that assesses attention and concentration, executive functions, memory, language, visuoconstructional skills, conceptual thinking, calculations, and orientation. An additional point is added for individuals with less than 12 years of education. Cognitive impairment is determined by an MoCA score of 25 points or below (Nasreddine et al. 2005; Arba et al. 2018). In our study, patients with acute ischemic stroke and symptoms of cognitive dysfunction for a minimum of three months following stroke were included in the PSCI group, and the remaining were enrolled in the post‐stroke non‐cognitive impairment (PSNCI) group.

### 2.4 Prediction Modeling and Statistical Analyses

A total of 261 patients with acute ischemic stroke were randomly assigned to the training dataset (*n* = 183) and testing dataset (*n* = 78) in a ratio of 7:3. Sensitive predictor variables were selected using the package LassoCV (sklearn.linear_model.LassoCV) in R. Two prediction modeling methods, namely the XGBoost (XGBoost library) and LR (sklearn.linear_model.LogisticRegression), were constructed to identify risk factors for PSCI. The accuracy, F1 score, Cohen's kappa, sensitivity, specificity, positive predictive value (PPV), and negative predictive value (NPV) were measured to compare the two prediction models. Receiver operating characteristic (ROC) curves were plotted to assess the prediction models’ ability to discriminate PSCI from PSNCI, and the area under the curve (AUC), with the corresponding 95% confidence interval (CI), was calculated via the bootstrap approach. Calibration curves, as generated by the package sklearn.calibration, displayed the consistency between the predicted and actual risks of PSCI. Decision curve analysis (DCA) assessed the changing trend of net benefit with the threshold and compared results for “intervention for all” versus “intervention for none.” True positive (TP), true negative (TN), false positive (FP), and false negative (FN) for each predicted threshold were calculated using user‐defined functions. The output of the XGBoost model was explained via SHapley Additive exPlanations (SHAP). In the training dataset, the SHAP value of each sample was quantified by the TreeExplainer algorithm. Feature importance contributed to the predictive ability of the XGBoost model, and the process of individual predictions was visualized in beeswarm and force plots, respectively.

Data were processed and visualized using R 4.4.1 and Python 3.8. Baseline characteristics were compared using the package CBCgrps in R. Measurement data in a normal distribution were expressed as mean ± standard deviation ($\bar{x}$± s), and compared by the *t*‐test between groups. Those in a skewed distribution were expressed as median and quartile, and compared by the Mann‐Whitney U test. Enumeration data, as expressed by *n* (%), were compared by the chi‐square test. *P* < 0.05 suggested a significant difference.

## 3 Results

### 3.1 Baseline Characteristics

A total of 261 patients with acute ischemic stroke were randomly assigned to the training dataset (*n* = 183) and testing dataset (*n* = 78) in a ratio of 7:3. The incidence of PSCI (66.7% [122/183] vs. 69.2% [54/78]) and other baseline characteristics were comparable between the two datasets (all *P* > 0.05) (Table 1).

**TABLE 1:** Baseline characteristics of patients with acute ischemic stroke (*n* = 261).

Variables	Total (*n* = 261)	Testing dataset (*n* = 78)	Training dataset (*n* = 183)	*p* value	Statistic
**Demographic data**					
Sex, *n* (%)	—	—	—	0.898	0.016
Male	191 (73)	58 (74)	133 (73)	—	—
Female	70 (27)	20 (26)	50 (27)	—	—
Age (years), median (Q1, Q3)	67 (60, 74)	69 (63.25, 74.75)	67 (59, 73.5)	0.095	8070
BMI (kg/m^2^), mean ± SD	24.5 ± 3.6	24.95 ± 3.96	24.31 ± 3.42	0.215	1.246
Education (years), mean ± SD	9 (6, 12)	9 (9, 12)	9 (6, 12)	0.236	1.185
**Clinical data**					
Smoking, *n* (%)	182 (70)	54 (69)	128 (70)	1	0
Drinking, *n* (%)	67 (26)	17 (22)	50 (27)	0.435	0.61
DM, *n* (%)	152 (58)	47 (60)	105 (57)	0.768	0.087
Hypertension, *n* (%)	47 (18)	15 (19)	32 (17)	0.873	0.026
**Cognitive function**					
PSCI, *n* (%)	176 (67)	54 (69)	122 (67)	0.795	0.068
NIHSS, median (Q1, Q3)	2 (1, 4)	2 (1, 4)	2 (1, 5)	0.125	6288.5
MoCA, median (Q1, Q3)	21 (14, 26)	22 (16.5, 26)	20 (13, 27)	0.582	0.551
ADL, median (Q1, Q3)	30 (21, 57)	29 (21, 52.5)	31 (21, 59)	0.51	6770
**Laboratory testing**					
VE‐Cad, median (Q1, Q3)	13.85 (11.37, 16.53)	13.71 (10.93, 15.51)	14.2 (11.46, 17.21)	0.264	6512.5
FGF21, median (Q1, Q3)	108.11 (64.87, 229.59)	108.11 (63.72, 211.34)	111.24 (67.13, 229.59)	0.447	6712
CRP, median (Q1, Q3)	1.3 (0.5, 3.51)	1.97 (0.5, 4.35)	1.22 (0.5, 3.07)	0.315	7694.5
TCH, median (Q1, Q3)	4.15 (3.5, 4.8)	4.16 (3.15, 4.5)	4.12 (3.56, 4.92)	0.232	6469.5
TG, median (Q1, Q3)	1.37 (0.93, 1.98)	1.32 (0.95, 1.76)	1.38 (0.92, 2.01)	0.387	6653.5
HDL‐C, median (Q1, Q3)	1.11 (0.92, 1.37)	1.1 (0.9, 1.34)	1.11 (0.92, 1.38)	0.55	6803
LDL‐C, median (Q1, Q3)	2.61 (2.03, 3.33)	2.72 (2, 3.16)	2.57 (2.04, 3.38)	0.473	6736
HCY, median (Q1, Q3)	12 (7.4, 15.4)	12.45 (9.4, 15.28)	11.7 (6.66, 15.45)	0.209	7836.5
UA, median (Q1, Q3)	310 (255, 378)	330 (260.75, 392.25)	309 (253.5, 369)	0.312	7702
HbA1c, median (Q1, Q3)	6.3 (5.7, 7.2)	6.05 (5.7, 6.9)	6.3 (5.7, 7.4)	0.313	6573.5
BUN/SCr ratio, median (Q1, Q3)	19.26 (15.49, 22.83)	19.39 (15.92, 23.28)	18.74 (15.42, 22.31)	0.58	7446.5
**Stroke circulation distribution, *n* (%)**	—	—	—	—	—
Anterior circulation stroke	155(59.4)	46(59.0)	109(59.6)	0.93	0.01
Posterior circulation stroke	71(27.2)	21(26.9)	50(27.3)	0.95	0.00
Mixed circulation stroke	35(13.4)	11(14.1)	24(13.1)	0.83	0.05
Location of stroke lesions, *n* (%)	—	—	—	—	—
Cortical involvement	74(28.4)	22(28.2)	52(28.4)	0.97	0.00
Subcortical/deep infarction	70(26.8)	21(26.9)	49(26.8)	0.98	0.00
Mixed cortical and subcortical infarction	53(20.3)	16(20.5)	37(20.2)	0.96	0.00
Brainstem/cerebellum	64(24.5)	19(24.4)	45(24.6)	0.97	0.00

Abbreviations: Q1, the first quartile; Q3, the third quartile; BMI, body mass index; SD, standard deviation; DM, diabetes mellitus; PSCI, post‐stroke cognitive impairment; NIHSS, the National Institutes of Health Stroke Scale; MoCA, Montreal Cognitive Assessment; ADL, the Activities of Daily Living; VE‐Cad, vascular endothelial cadherin; FGF21, fibroblast growth factor 21; CRP, C‐reactive protein; TCH, total cholesterol; TG, triglyceride; HDL‐C, high‐density lipoprotein cholesterol; LDL‐C, low‐density lipoprotein cholesterol; HCY, homocysteine; UA, uric acid; HbA1c, hemoglobin A1c; BUN, blood urea nitrogen; SCr, serum creatinine.

*Note*: Measurement data were first tested for the normality using the Shapiro‐Wilk test, and normally distributed data were expressed as mean ± standard deviation (±s), and analyzed between groups using the independent samples t test; otherwise, they were expressed as the median (interquartile range) (M [IQR]), and analyzed via the Mann‐Whitney U test. Enumeration data, as expressed as the number of cases (percentage) (*n*%), were analyzed using the chi‐square test or Fisher's exact test (expected frequency of less than 5). *P* < 0.05 considered as statistically significant.

### 3.2 Variable Selection via Lasso Regression

**FIGURE 1:**
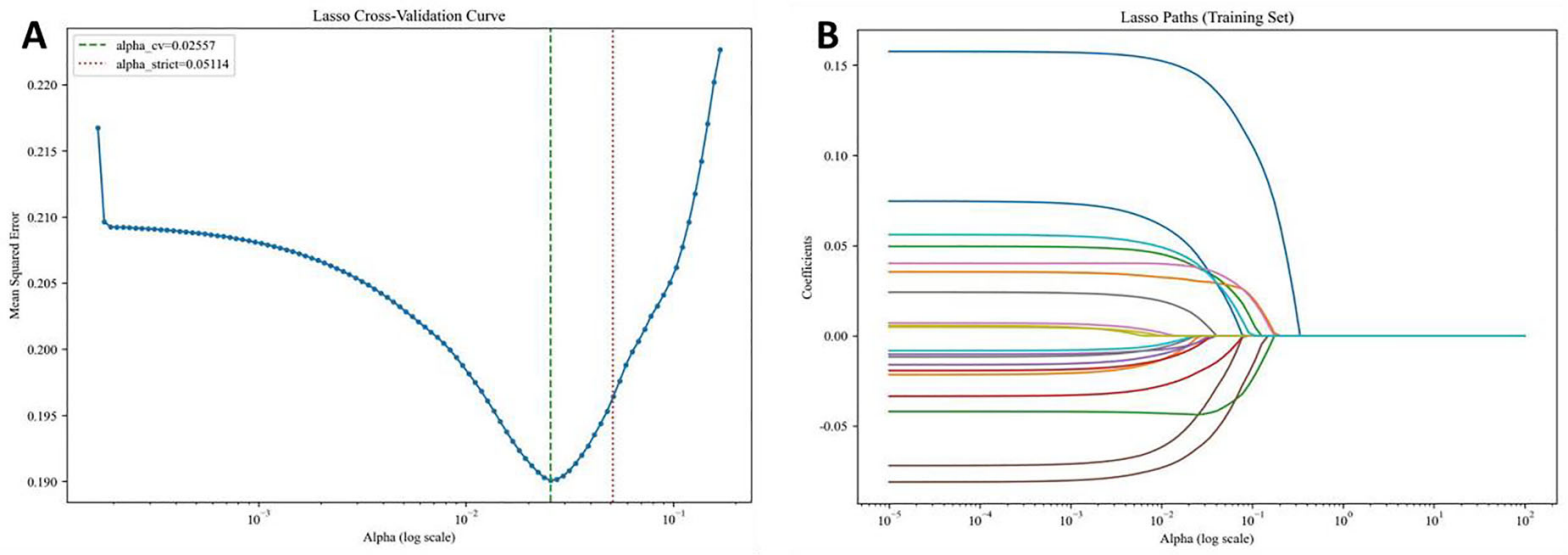
Lasso paths (A) and cross‐validation curve (B) for variable regularization and selection.

All potential risk factors for PSCI, as described in Table 1, were selected via Lasso regression using the package LassoCV. The optimal regularization parameter alpha (α) was automatically selected by the 10‐fold cross validation, and all variables with a non‐zero coefficient were retained for the following modeling. Lasso paths (Figure 1A) and cross‐validation curves (Figure 1B) directly visualized the process of variable regularization and selection. Finally, a box of sensitive variables was identified, including VE‐Cad, NIHSS score, CRP, age, drinking history, and education years, for assessing the efficacy of the prediction models.

### 3.3 XGBoost and LR Models for Predicting the Risk of PSCI

Two prediction models, as created by XGBoost and LR, were used to identify risk factors for PSCI. Their performance was later assessed in the testing dataset.

**FIGURE 2:**
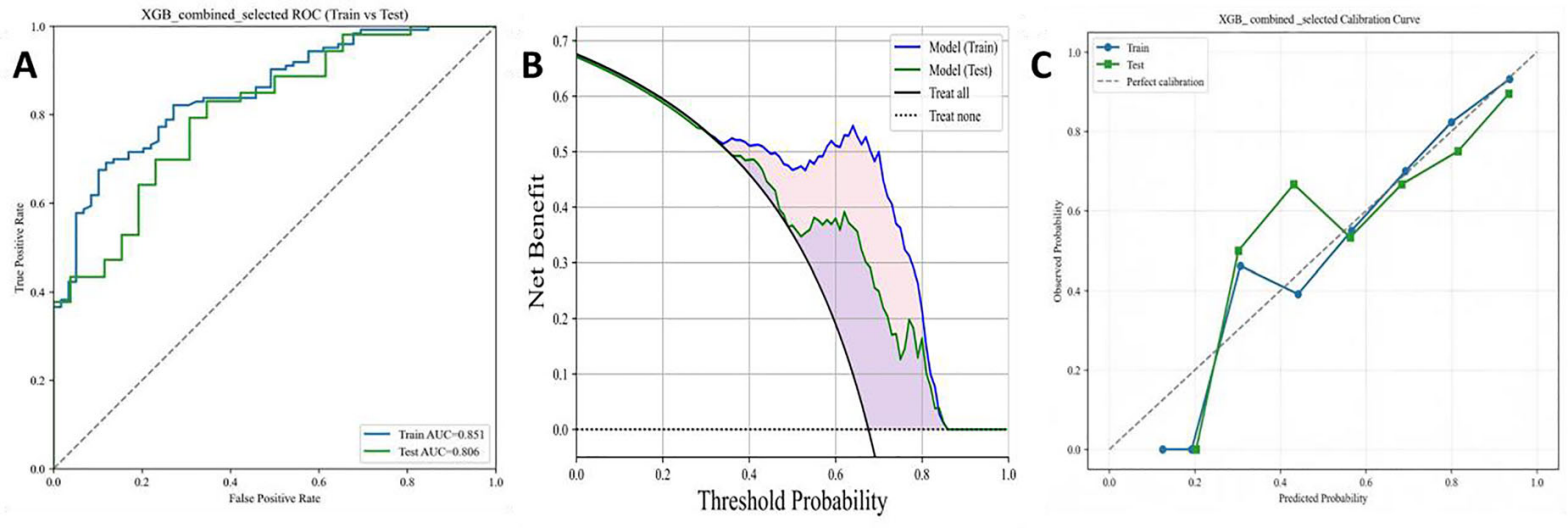
ROC (A), DCA (B) and calibration curves (C) of the XGBoost model in predicting the risk of PSCI in training and testing datasets.

The XGBoost model yielded an AUC of 0.851 (95% CI, 0.791–0.902) in the training dataset, and 0.806 (95% CI, 0.705–0.898) in the testing dataset (Figure 2A). The sensitivity was 90.2% and 84.9% in the training and testing datasets, respectively. The F1 score was 84.4 in the training dataset, and 81.1 in the testing dataset (Table 2). All these values suggested a high efficiency of the XGBoost model in discriminating PSCI from PSNCI. DCA proved the clinical usefulness of the XGBoost model in predicting the risk of PSCI, where the x‐axis denoted the threshold probability and the y‐axis was the net benefit between TP (benefit from the intervention) and FP (harms of the inappropriate intervention). As shown in Figure 2B, the grey solid line represented the “treat all” strategy, suggesting zero net benefit in treating all individuals. The black solid line parallel to the x‐axis described the “treat none” strategy. Additionally, the calibration curve plotted good consistency between the predicted probabilities and observed frequencies in both datasets, indicating a high‐level determinatory ability (Figure 2C).

**TABLE 2:** Metrics assessing the performance of the XGBoost model in predicting the risk of PSCI.

Metrics (95% CI)	Testing dataset (*n* = 78)	Training dataset (*n* = 183)
Accuracy	0.73 (0.65, 0.82)	0.77 (0.71, 0.83)
F1 score	0.81 (0.73, 0.88)	0.84 (0.79, 0.89)
Cohen's kappa	0.20 (−0.01, 0.44)	0.43 (0.30, 0.56)
Sensitivity	0.85 (0.75, 0.94)	0.90 (0.85, 0.95)
Specificity	0.50(0.31, 0.69)	0.51 (0.37, 0.64)
PPV	0.78 (0.67, 0.88)	0.79 (0.72, 0.86)
NPV	0.62 (0.41, 0.83)	0.71 (0.57, 0.85)

Abbreviations: XGBoost, extreme gradient boosting; PSCI, post‐stroke cognitive impairment; CI, confidence interval; PPV, positive predictive value; NPV, negative predictive value.

*Note*: Then, the performance of LR model was assessed.

**FIGURE 3:**
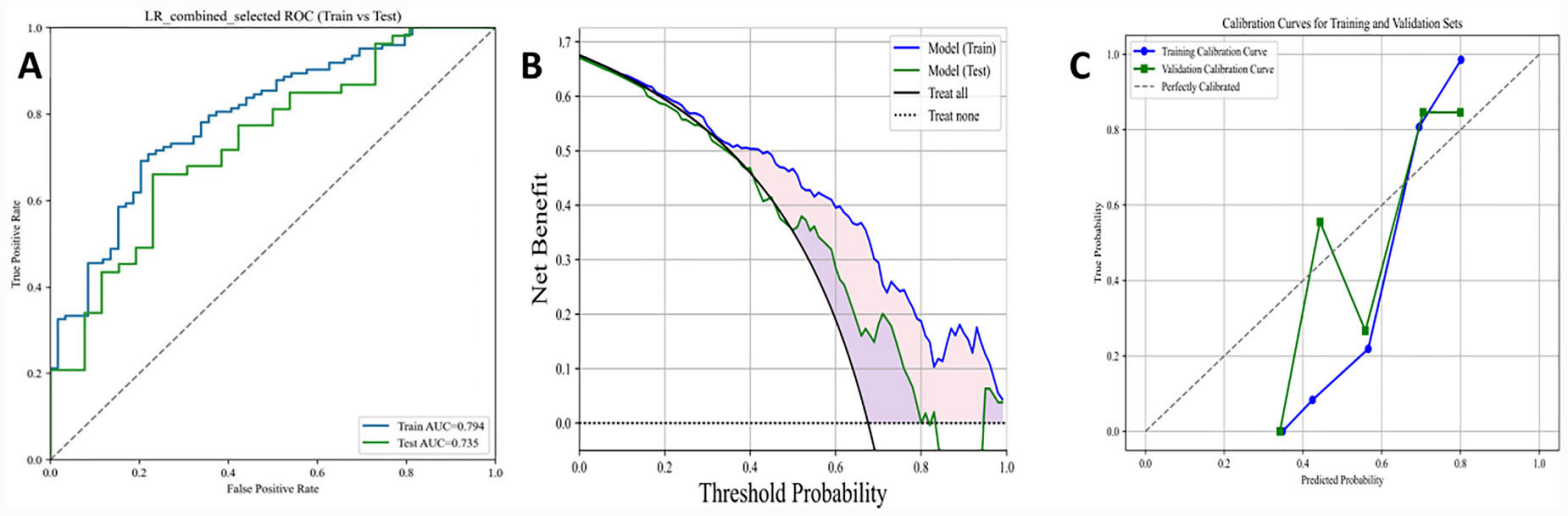
ROC (A), DCA (B) and calibration curves (C) of the LR model in predicting the risk of PSCI in training and testing datasets.

Then, the performance of the LR model was assessed. The LR model yielded an AUC of 0.794 (95% CI, 0.724–0.857) in the training dataset, and 0.735 (95% CI, 0.622–0.843) in the testing dataset (Figure 3A). DCA showed its potential for practical application (Figure 3B), but the calibration curve indicates that the model is not generalized enough (Figure 3C). The sensitivity was 87.8% and 84.9% in the training and testing datasets, respectively. The F1 score was 77.3 in the training dataset, and 71.7 in the testing dataset (Table 3).

**TABLE 3:** Metrics assessing the performance of the LR model in predicting the risk of PSCI.

Metrics (95% CI)	Testing dataset (*n* = 78)	Training dataset (*n* = 183)
Accuracy	0.68 (0.58, 0.78)	0.79 (0.73, 0.85)
F1 score	0.72 (0.61, 0.82)	0.77 (0.71, 0.83)
Cohen's kappa	0.25 (0.02, 0.50)	0.49 (0.34, 0.63)
Sensitivity	0.85 (0.76, 0.94)	0.88 (0.82, 0.93)
Specificity	0.35 (0.17, 0.53)	0.49 (0.36, 0.61)
PPV	0.74 (0.61, 0.83)	0.81 (0.71, 0.83)
NPV	0.52 (0.27, 0.88)	0.73 (0.85, 1.00)

Abbreviations: LR, logistic regression; PSCI, post‐stroke cognitive impairment; CI, confidence interval; PPV, positive predictive value; NPV, negative predictive value.

### 3.4 Visualization of XGBoost and LR Models in Predicting the Risk of PSCI

**FIGURE 4:**
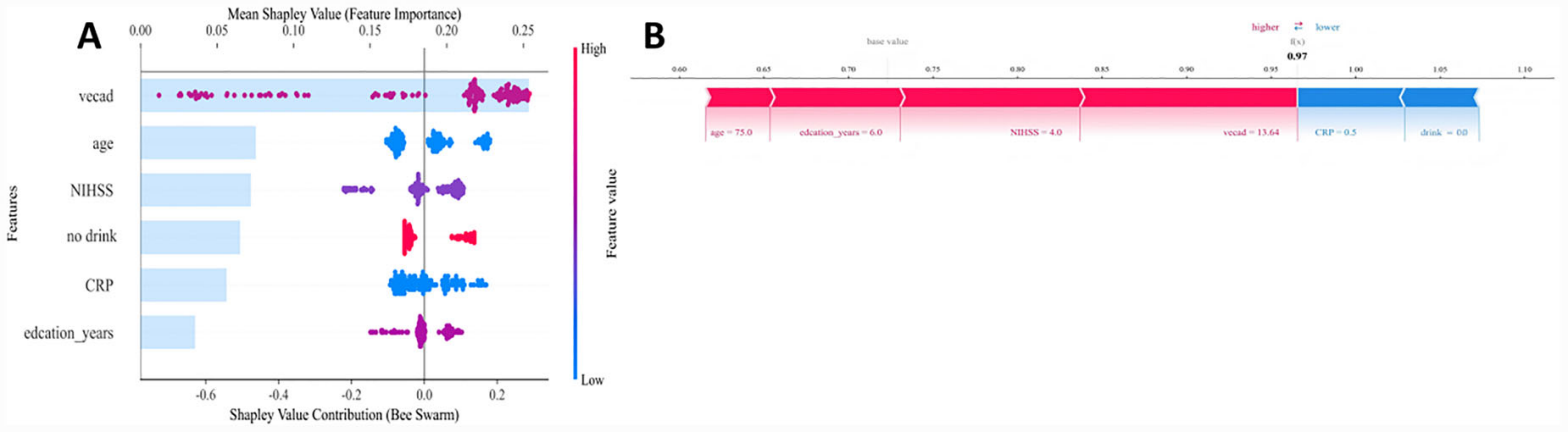
SHAP beeswarm (A) and force plots (B) visualizing the XGBoost model in predicting the risk of PSCI.

Superior to the LR model, the XGBoost model graded better in the AUC (0.806 vs. 0.735), F1 score (81.1 vs. 71.7), and sensitivity (84.9% vs. 84.9%) in the testing dataset, indicating stronger potential of the machine learning technique of gradient boosting in predicting PSCI. A cross validation of the two algorithms further enhanced the credibility of the XGBoost model's effectiveness. SHAP beeswarm plots clearly visualized the impact of each feature in the XGBoost model on the predicted risk of PSCI (Figure 4A), where the horizontal axis represented SHAP values, and the vertical axis listed all features sorted by the degree of influence of cumulative SHAP values. Specifically, red dots denoted high‐value features, while blue dots low‐value features. In our study, VE‐Cad, NIHSS score, CRP, age, drink, and education years were predominant features influencing the onset of PSCI in patients with acute ischemic stroke.

In addition, SHAP force plots displayed feature contributions to the predicted risk of PSCI from left (features with positive contributions labeled in red) to right (features with negative contributions labeled in blue), with the base value as the reference. The strength of contribution was qualified by the magnitude of the SHAP value (length of the bar). Force plots quantified the influence of each feature in the XGBoost model on the predicted risk of PSCI in patients with acute ischemic stroke and visualized the process of individual prediction. Here, serum VE‐Cad, as labeled in red, significantly increased the risk of PSCI when exceeding 13.64 ng/mL. CRP was labeled in red, suggesting that the risk of PSCI greatly increased when CRP levels exceeded 10.27 mg/L. On the contrary, an NIHSS score of 4 or below, and non‐drinking, as labeled in blue, showed negative contributions to the predicted risk of PSCI, suggesting that they were protective factors.

### 3.5 Model Performance Comparisons to Quantify the Incremental Value of Serum Markers for Predicting PSCI

A comparison of the XGBoost model (involvement of serum markers and demographic and clinical variables) and the baseline clinical model (involvement of only demographic and clinical variables) was made to quantify the incremental value of serum markers for predicting PSCI. The XGBoost model earned a net AUC enhancement of 0.178 than the baseline clinical model (0.806 [95% CI, 0.705–0.898] vs. 0.628 [95% CI, 0.552–0.798]) in the testing dataset (Table 4). Additionally, a higher sensitivity of the XGBoost model than the baseline clinical model suggested the additional effects of serum markers in more effectively recognizing high‐risk individuals with PSCI (85.0% vs. 60.4%). The two prediction models presented a similar ability (specificity, 50.0% vs. 54.2%) in discriminating non‐PSCI individuals from PSCI patients.

**TABLE 4:** Comparison of performance between the full model and the baseline clinical model on the validation set.

Model	AUC_mean	Sensitivity (%)	Specificity (%)	Accuracy (%)	F1 score
LR_baseline_clinical	0.664	67.9	61.5	65.8	64.3
XGB_baseline_clinical	0.628	60.4	54.2	67.1	65.2
LR_combined_selected	0.735	84.9	34.6	68.4	71.7
XGB_combined_selected	0.806	85.0	50	73.4	81.1

## 4 Discussion

### 4.1 Clinical Manifestations of PSCI

Cognitive impairment occurs in more than 50% of stroke patients within 3 months of disease onset (Lo et al. 2019; Kalaria et al. 2016). Consistently, the incidence of PSCI was 67.4% in our cohort of 261 patients with acute ischemic stroke. The higher incidence in our cohort than the previously reported upper limit of the range could be attributed to the assessment of cognitive impairment within three months of stroke using the MoCA, a highly sensitive screening instrument, as well as the allocation of a higher‐risk population of hospitalized acute ischemic stroke patients. PSCI, as a common but severe after‐effect, undermines the quality of life and social participation. Moreover, both physiological and psychological rehabilitation can be greatly decelerated by PSCI, posing huge mental and economic burdens on affected people and their family members (Xue et al. 2021). Epidemiological data show that the 5‐year mortality is 41% higher in stroke patients with PSCI than in those with PSNCI (Kapoor et al. 2019). PSCI can progressively and irreversibly progress into dementia without effective interventions. Consequently, early recognition of PSCI prior to the development of dementia is the best way to improve the prognosis of PSCI.

Demographic data and neuroimaging indicators are frequently used in generating prediction models for PSCI. However, the obtainment of neuroimaging data is limited by high costs, strict indications, and lack of unified standards. Here, we incorporated easily accessible, low‐cost, and repeatable PSCI‐related serum markers into machine learning prediction models. In an integration of both common demographic and clinical data, as well as serum markers, XGBoost and LR models were created. Superior to the LR model, the XGBoost model discriminated PSCI from PSNCI with an AUC of 0.851 (95% CI, 0.791–0.902) in the training dataset and 0.806 (95% CI, 0.705–0.898) in the testing dataset. DCA and calibration curves also proved the better performance of the XGBoost model than the LR model. In addition, SHAP identified that serum VE‐Cad was the feature contributing the largest to the prediction in the XGBoost model, followed by age, NIHSS score, drink, CRP, and education years.

### 4.2 Factors Influencing the Risk of PSCI

#### 4.2.1 VE‐Cad

VE‐Cad is a calcium‐dependent, cell‐cell adhesion, endothelial‐specific molecule mainly expressed at the cell junctions of brain microvascular endothelial cells. The binding of VE‐Cad to catenins is essential for maintaining the permeability and integrity of vascular endothelial cells and regulating BBB function (Nakano‐Doi et al. 2018). The BBB, as a physiological barrier between the central nervous system and peripheral blood circulation, protects the brain from toxic substances and inflammatory factors in the blood while allowing essential nutrients to pass through (Benz and Liebner 2022). Impaired BBB integrity is an established pathogenic factor for PSCI in ischemic stroke patients (Prakash and Carmichael 2015; Panahpour et al. 2018; Jin et al. 2010). A two‐year follow‐up study showed that higher BBB leakage volume and leakage rate indicate worse cognitive decline (Kerkhofs et al. 2021). In response to pathological stimuli (e.g., ischemia, hypoxia, inflammation, stress), a variety of proteases like matrix metalloproteinases (MMPs) are activated to cut off the extracellular domain of VE‐Cad at cell junctions, leading to the disruption of intercellular connections and the breakdown of the extramembrane portion. The formation of N‐based residues eventually results in the elevation of serum soluble VE‐Cad, causing the integrity damage and increased permeability of the BBB (Demeyere and Moore 2024). Consequently, the abnormally elevated VE‐Cad aggravates the BBB damage through the following mechanisms. First, the increased VE‐Cad directly enhances gaps between endothelial cells by destabilizing VE‐Cad complexes through impairing binding affinities (Wu et al. 2025). Second, intracellular signals, such as the Wnt‐β‐catenin and others that disrupt the BBB are activated by VE‐Cad. Under normal circumstances, β‐catenin forms a complex with VE‐Cad anchored on the endothelial cell membrane. The increased VE‐Cad disrupts the complex and dissociates β‐catenin at the junction site, leading to the imbalance of signal transductions that protect the integrity of the BBB (Zhong et al. 2025). Third, the inhibitory effect of the intact VE‐Cad complex against inflammatory responses is impaired by the elevated VE‐Cad. As a result, abundant inflammatory factors and adhesion molecules are activated to recruit leukocyte adhesion and migration (Wang and Dong 2021). Overall, the increase in VE‐Cad levels indicates a persistent condition of impaired stability and imbalanced repair of endothelial cell connections and BBB integrity in PSCI patients, indirectly reflecting the severity of BBB damage and the pathological degree of PSCI. In the present study, VE‐Cad possessed the highest SHAP value among sensitive features in predicting the risk of PSCI, emphasizing its dominant role in the XGBoost model. SHAP force plots further displayed the risk of PSCI increasing with VE‐Cad levels. The risk of PSCI greatly increased when VE‐Cad levels exceeded 13.64 ng/L.

#### 4.2.2 CRP

CRP, a plasma protein synthesized by the liver, increases sharply in response to infections or tissue damage, serving as a non‐specific biomarker for infections. CRP induces the uptake of low‐density lipoproteins by macrophages to form foam cells, thereby impairing endothelial function or inducing abnormal migration and proliferation of vascular smooth muscle cells (Zhang and Bi 2020). Hence, CRP is a risk factor for cerebral atherosclerosis, stroke, and other cerebrovascular lesions (Boncler et al. 2019). CRP infiltrates the ischemic regions of the brain when the BBB integrity is impaired following ischemic stroke. Co‐localized with p‐Tau and amyloid‐beta (Aβ), as well as spatially overlapped with CD68^+^ microglia and IL‐1β^+^ inflammatory areas, CRP in the monomeric form (mCRP) deposits in the infarct area and surrounding tissues, indicating its involvement in the interplay between chronic neuroinflammation and neurodegeneration (Al‐Baradie et al. 2021). Additionally, the binding of mCRP to TLR4 drives microglial polarization toward the M1 pro‐inflammatory phenotype and release of IL‐1β, TNF‐α, IL‐6, and other cytokines via activating the MyD88‐NF‐κB signaling pathway, forming an amplified inflammatory cascade (Șalari and Slevin 2025). CRP also directly induces neuronal apoptosis by generating C5b‐9, also known as the membrane attack complex, through activating the classical complement pathway (Pastorello et al. 2023). Therefore, mCRP, a key mediator of local inflammation, contributes to widespread brain damage by promoting microvascular instability, neuroinflammation, and neuronal degeneration. Its active expression in distal brain regions (e.g., the hypothalamus) further highlights its potential to propagate inflammatory cascades, leading to long‐term cognitive decline and aggravation of PSCI (Jeon et al. 2021). Notably, our findings uncovered the synergistic effect of CRP and VE‐Cad on predicting PSCI, and the underlying molecular mechanism. The elevated peripheral blood CRP dissociates into mCRP in response to the local ischemia, which further specifically binds to CD31 on the surface of endothelial cells, triggers CD31 phosphorylation, and weakens the binding affinity with VE‐Cad (Șalari and Slevin 2025; Kim et al. 2022). Meanwhile, the suppressed activity of protein phosphatase 2 (PP2A) within an inflammatory environment causes VE‐Cad phosphorylation on Ser665, directly stimulating the internalization and degradation of VE‐Cad. This post‐translational modification also fosters a vicious cycle with the mCRP‐CD31 axis that further exposes the phosphorylation site of VE‐Cad through impairing the adherens junction, allowing a preference of VE‐Cad internalization. Consequently, the loss of VE‐Cad aggravates the leakage of mCRP into the brain parenchyma (Shaw et al. 2001). An “outside‐in” molecular priming superimposed over an “inside‐out” complex disassembly synergistically amplifies the impaired integrity of the BBB, neuroinflammatory infiltration, and the subsequent cognitive function impairment, pathologically bridging peripheral inflammatory markers with neurodegeneration (Guo et al. 2018). In our XGBoost model, CRP was the third contributive feature in predicting the risk of PSCI in patients with acute ischemic stroke. The risk of PSCI greatly increased when CRP levels exceeded 10.27 mg/L.

### 4.3 Other Factors

In our prediction model, baseline age, NIHSS score, drinking, and education years were all associated with the risk of PSCI.

The volume and mass of the brain gradually decline with aging, and the progressive loss of neurons in the gray matter and hippocampus eventually accelerates the development of PSCI (Wang et al. 2024).

Long‐term alcohol consumption increases the risk of PSCI through multiple mechanisms. Alcohol and its metabolite acetaldehyde exhibit direct neurotoxicity, inducing oxidative stress and excitotoxicity, which leads to neuronal damage in critical brain regions such as the hippocampus. Additionally, alcohol consumption disrupts the BBB, exacerbates neuroinflammation, and impairs cognitive functional connectivity by inducing cerebral microbleeds and white matter damage (Laari et al. 2024). Furthermore, the frequent deficiency of vitamin B1 associated with alcohol use may further aggravate memory impairment (Ben et al. 2021).

The NIHSS assesses the severity of neurological deficits post‐stroke, and its value is correlated with the volume of infarction lesions. Stroke patients with high NIHSS scores usually show large‐scale infarction or infarction in critical brain regions, manifested as severe motor or language dysfunction. Moreover, a larger infarction spanning multiple brain regions is prone to cognitive deterioration and even dementia (Rundek et al. 2022).

Years of education serve as a crucial protective factor against PSCI, with its core mechanism rooted in cognitive reserve. Higher education enhances the efficiency and plasticity of neural networks in the brain, enabling individuals to more effectively activate alternative neural networks for compensation when facing stroke‐induced brain damage. This compensatory capacity acts as a “buffer” for the brain, allowing individuals with higher education to maintain relatively better cognitive function even when experiencing identical pathological damage (Umarova et al. 2019).

### 4.4 Clinical Care Pathway for the Risk Stratification of PSCI

A clinical care pathway for the risk stratification of PSCI was proposed to translate the XGBoost model into clinical practice. Specifically, a three‐level risk threshold framework was determined by the DCA findings to categorize individuals into low‐risk (predicted probability < 62.6%), medium‐risk (62.6%–68.6%), and high‐risk groups of PSCI (>68.6%). Within 24 h of admission, the predicted probability was computed based on age, NIHSS scores, and laboratory testing of VE‐Cad, CRP, thus guiding the following stratified management. First, individuals with a low risk of PSCI were managed by secondary prevention and health education. Cognitive screening was recommended to this population at 6–12 months post‐discharge. Second, moderate‐risk patients were routinely treated with intensive blood pressure, blood sugar, and blood lipid management. Referred to the rehabilitation department, cognitive training could be initiated as early as possible. Follow‐up visits for cognitive assessment were provided at 3 and 6 months. Third, a multidisciplinary team involving physicians in the departments of neurology, rehabilitation, and clinical psychology was engaged in formulating an individualized, highly intensive, multi‐modal cognitive recovery plan to manage individuals with a high risk of PSCI. Their family members were also educated with medical knowledge about PSCI. Superior to the conventional treatment that PSCI patients have passively responded to cognitive decline, this stratified management based on the XGBoost model looks ahead at what may happen and is ready to handle it. Moreover, all variables involved in the XGBoost model were highly accessible and routinely detected with low costs. This prediction model is expected to be embedded in the electronic medical record system, thereby achieving the goal of automatic risk calculation and real‐time warning.

### 4.5 Considerations of the Validation Strategies in Predictive Models and Future Directions

In this single‐center, preliminary, exploratory clinical trial, we aim to screen potential serum markers and create a series of models to predict the risk of PSCI. Their performance was assessed by a combination strategy involving both an internal verification and strict methodological control through four aspects. By a random split into the training and testing datasets, the prediction performance was first validated in non‐modified independent samples. Second, a 10‐fold cross‐validation allowed the stability of the selected variables. Third, a comparative verification of the XGBoost and LR models with distinct algorithms was made to enhance the credibility of the predictive modeling. Fourth, the prediction model was validated from the three dimensions of discrimination, calibration, and clinical utility. Altogether, these validation strategies allowed the rigor of model construction and the reliability of prediction results. However, an external validation in independent samples from other centers or over different time periods was not applied to our prediction models, which can be crucial to assess the generalizability of the prediction model across populations and clinical settings. In the future, time‐series prospective validation (a prospective cohort allocated in our medical institution to validate the model stability across time) and multi‐center spatial validation (an external validation in participants from other medical institutions and those with distinct demographic and regional characteristics) will be the two research focuses to further strengthen the versatility and clinical promotion value of the XGBoost model in predicting PSCI.

### 4.6 Limitations

This study had the following limitations. First, this was a single‐center study with a relatively small sample size (*n* = 261), involving patients predominantly from East China. Multi‐center external validation is required to assess the universality of the prediction model across other regions and medical institutions. Second, pre‐stroke cognitive assessment was mainly through retrospective interviews with caregivers and inquiry of medical records, although following the guidance of the Informant Questionnaire on Cognitive Decline in the Elderly (IQCODE), may potentially lead to retrospective biases and be less sensitive to recognizing the very mild prodromal cognitive impairment. Third, serum markers were examined at a single time point within 24 h of onset, which barely reflected the dynamic trajectory and temporal changes in PSCI. Fourth, imaging indicators of PSCI, such as infarct volume, lesion location, and the TOAST (Trial of Org 10172 in Acute Stroke Treatment) classification of stroke have not been examined. While serum markers are easily accessible in case of limited medical records, the lack of imaging markers inevitably influences the strength of the predictive modeling. Finally, we did not analyze genetic indicators (the APOE genotyping) in post‐stroke patients. The potential of genetic susceptibility in predicting the risk of PSCI should be further explored. In the future, more multi‐center, large‐scale prospective studies involving imaging and genetic indicators that are dynamically monitored are essential to promote clinical translation and application of the prediction model, thus benefiting the early recognition and management of PSCI.

## 5 Conclusion

In this study, we constructed and validated a cognitive impairment prediction model for acute ischemic stroke based on blood biomarkers. Machine learning algorithms were employed to develop LR and XGBoost models, with the latter demonstrating superior performance. SHAP analysis revealed that VE‐Cad level, NIHSS score, CRP, age, drinking, and education years were significant risk factors for cognitive impairment in patients with acute ischemic stroke. The XGBoost model incorporating these easily accessible indicators, as a supplement or alternative tool, is of clinical significance to assist in the rapid identification, early interventions, and guidance of treatment strategies against PSCI. Besides the basic predictive value of age and NIHSS score, we for the first time systematically verified the incremental value of a box of serum markers (VE‐Cad, CRP) in predicting PSCI. Notably, VE‐Cad, as a key indicator of BBB integrity, was the most eye‐catching variable in the XGBoost model. Its involvement in the prediction model overturned the conventional clinical assessment of PSCI, stepping into novel, real‐time, vascular‐specific monitoring.

Collectively, the XGBoost model created based on serum markers is a valuable tool to predict the risk of PSCI. Moreover, the exact role of screened serum markers, either as biomarkers or pathogenic mediators of PSCI, and data mining of their targets are future research spotlights.

## Author Contributions

**Yang Xiaofeng** carried out the research design, analyzed data and wrote the main manuscript. **Li Xianwen** guided the research and critically reviewed the work. **Wu Yanfeng** acquired funding and contributed to data collection. **Zhang Qian**, **Chen Juan** and **Zhao Fengjiao** performed scale assessments and data collection. All authors read and approved the final manuscript.

## Funding

This work was supported by the Jiangsu Provincial Health Commission 2021 Medical Research Project (NO.M2021064).

## Ethics Statement

The experimental procedures were all in accordance with the guideline of the Ethics Committee of The Second Affiliated Hospital of Nanjing Medical University.

## Consent

A signed written informed consent was provided by all participants or their family members.

## Conflicts of Interest

The authors declare no conflicts of interests.

## Data Availability

The data used and analyzed can be obtained from the corresponding author under a reasonable request.
